# Extended-Spectrum-Beta-Lactamases, AmpC Beta-Lactamases and Plasmid Mediated Quinolone Resistance in *Klebsiella* spp. from Companion Animals in Italy

**DOI:** 10.1371/journal.pone.0090564

**Published:** 2014-03-04

**Authors:** Valentina Donati, Fabiola Feltrin, Rene S. Hendriksen, Christina Aaby Svendsen, Gessica Cordaro, Aurora García-Fernández, Serena Lorenzetti, Raniero Lorenzetti, Antonio Battisti, Alessia Franco

**Affiliations:** 1 Istituto Zooprofilattico Sperimentale delle Regioni Lazio e Toscana, Rome, Italy; 2 Technical University of Denmark, National Food Institute (DTU-Food), Kongens Lyngby, Denmark; 3 Istituto Superiore di Sanità, Department of Infectious, Parasitic and Immune-Mediated Diseases, Rome, Italy; Institut National de la Recherche Agronomique, France

## Abstract

We report the genetic characterization of 15 *Klebsiella pneumoniae* (KP) and 4 isolates of *K. oxytoca* (KO) from clinical cases in dogs and cats and showing extended-spectrum cephalosporin (ESC) resistance. Extended spectrum beta-lactamase (ESBL) and AmpC genes, plasmid-mediated quinolone resistance (PMQR) and co-resistances were investigated. Among KP isolates, ST101 clone was predominant (8/15, 53%), followed by ST15 (4/15, 27%). ST11 and ST340, belonging to Clonal Complex (CC)11, were detected in 2012 (3/15, 20%). MLST on KP isolates corresponded well with PFGE results, with 11 different PFGE patterns observed, including two clusters of two (ST340) and four (ST101) indistinguishable isolates, respectively. All isolates harbored at least one ESBL or AmpC gene, all carried on transferable plasmids (IncR, IncFII, IncI1, IncN), and 16/19 were positive for PMQR genes (*qnr* family or *aac(6′)-Ib-cr*). The most frequent ESBL was CTX-M-15 (11/19, 58%), detected in all KP ST101, in one KP ST15 and in both KP ST340. *bla*_CTX-M-15_ was carried on IncR plasmids in all but one KP isolate. All KP ST15 isolates harbored different ESC resistance genes and different plasmids, and presented the non-transferable *bla*_SHV-28_ gene, in association with *bla*_CTX-M-15_, *bla*_CTX-M-1_ (on IncR, or on IncN), *bla*_SHV-2a_ (on IncR) or *bla*_CMY-2_ genes (on IncI1). KO isolates were positive for *bla*_CTX-M-9_ gene (on IncHI2), or for the *bla*_SHV-12_ and *bla*_DHA-1_ genes (on IncL/M). They were all positive for *qnr* genes, and one also for the *aac(6′)-Ib-cr* gene. All *Klebsiella* isolates showed multiresistance towards aminoglycosides, sulfonamides, tetracyclines, trimethoprim and amphenicols, mediated by *strA/B*, *aadA2*, *aadB*, ant (2*")-Ia*, *aac(6′)-Ib*, *sul*, *tet*, *dfr* and *cat* genes in various combinations. The emergence in pets of multidrug-resistant *Klebsiella* with ESBL, AmpC and PMQR determinants, poses further and serious challenges in companion animal therapy and raise concerns for possible bi-directional transmission between pets and humans, especially at household level.

## Introduction

*Klebsiella* are bacterial pathogens that can cause a variety of severe infections in humans, mainly due to *K. pneumoniae* (KP) [1], [2] and to a lesser degree to *K. oxytoca* (KO) [3], [4]. KP is also a well-known causal agent of mastitis in cattle and bacteraemia in calves, cervicitis and metritis in mares, pneumonia and septicemia in foals, pneumonia, urinary tract infection (UTI) and septicemia in dogs [5], [6], [7].

Increasing antimicrobial resistance, especially towards aminoglycosides, (fluoro)quinolones, third and fourth generation cephalosporins, cephamycins, and carbapenems have been reported in the last decade [8], [9], [10], and poses serious therapeutic problems when treating *Klebsiella* infections in humans. In veterinary medicine, scarce information is reported on the occurrence of extended spectrum beta-lactamases (ESBLs), AmpC beta-lactamases and plasmid mediated quinolone resistance (PMQR) in *Klebsiella* isolates from companion animals [11], [12].

The aim of the study was to provide molecular characterization of extended-spectrum cephalosporin (ESC) resistance and PMQR in *Klebsiella* isolates from clinical cases or lesions in necropsied animals of canine and feline origin in Italy. A further aim was to determine phenotype and genotype of co-resistances, and to provide plasmid identification and genetic relatedness by Multilocus Sequence Typing (MLST) and Pulsed Field Gel Electrophoresis (PFGE) among the isolates, to evaluate potential clustering of ESC, PMQR, and other resistance genes among clones.

## Materials and Methods

### Origin of ESC-resistant Klebsiella

Between 2006 and 2012, the Istituto Zooprofilattico Sperimentale delle Regioni Lazio e Toscana (IZSLT) investigated samples from 1555 dogs and 429 cats of clinical cases and necropsy specimens with suspicious bacterial infections, submitted by veterinarians practising mainly in central Italy, and some practising in northern Italy. Presumptive positive *Klebsiella* isolates were identified using the API 20E identification system (bioMérieux, Craponne, France). For species-level identification of isolates with phenotypic inconclusive results 16S rDNA sequencing technique was employed, by means of the MicroSeq Full Gene system (Applied Biosystems, USA) as described previously [13].

### Genotypic characterization

Multilocus Sequence Typing on KP isolates was performed as previously described [14], and interpreted according to the KP MLST database (www.pasteur.fr/mlst).

In addition, all isolates were genotyped by PFGE using *Xba*I according to the previously published protocol [15].

### Antimicrobial susceptibility testing

Antimicrobial susceptibility testing was performed as minimum inhibitory concentrations (MIC) by micro-broth dilution in 96-well microtitre plates (Trek Diagnostic Systems, Westlake, OH, USA). The following antimicrobials were tested: ampicillin, cefotaxime, ceftazidime, ciprofloxacin, chloramphenicol, florfenicol, gentamicin, kanamycin, nalidixic acid, streptomycin, sulfonamides, tetracycline, and trimethoprim. The results were interpreted according to the European Committee on Antibiotic Susceptibility Testing (EUCAST) epidemiological cut-offs (www.eucast.org) and to Clinical Laboratory Standard Institute [16] or EUCAST clinical breakpoints for those drugs for which epidemiological cut-offs have not been made available (kanamycin, chloramphenicol, sulfamethoxazole, trimethoprim). For streptomycin, a cut-off of 16 mg/L was used, according to EUCAST MIC distributions.

Confirmatory test for the detection of ESBLs were performed on isolates resistant to cefotaxime or ceftazidime according to Clinical Laboratory Standard Institute (CLSI) recommendations [16].

### Detection of genes encoding beta-lactamase and PMQR

For the confirmed ESBL producing isolates, the encoding genes belonging to the beta-lactamase and PMQR families were further analyzed for the presence of *bla*_CTX-M_ [17], *bla*_SHV_ [18], *bla*_TEM_ [19], *bla*_OXA_ [20], *bla*_AmpC_ families [21], as well as for genes of the *qnr* family, *qep*-A, and *aac(6′)-Ib-cr* encoding for PMQR [22], [23], [24], [25], [26]. The isolates were further screened by PCR for genes encoding carbapenemases [27]. Amplicons were sequenced by BigDye Terminator chemistry (Applied Biosystems, Foster City, CA, USA) and migrated with an automated sequencer (ABI Prism 310; Applied Biosystems). Sequence data analysis was performed using CLC DNA workbench software version 5.7.1 (CLC Bio, Aarhus, Denmark) and evaluated against the GenBank nucleotide databases.

### Detection of plasmid replicons

Identification of plasmids was performed by PCR-based replicon typing as previously described [28], [29], [30], and using the PBRT kit (Diatheva, Fano, Italy).

### Plasmid analysis

Plasmid DNA preparations were performed using the NucleoSpin Plasmid/Plasmid (NoLid) kit (Macherey-Nagel, Düren, Deutschland) and used to transform MAX Efficiency DH5α Competent Cells (Invitrogen, Life Technologies, U.S.A). In order to identify the plasmid carrying the ESBLs and AmpC genes, the selection of the transformants was performed on LB agar plates containing 100 µg/ml ampicillin.

Additionally, the isolates were tested according to the manufacturer's instructions using an array hybridization kit for DNA-based detection of the most common resistance genes, and for the integrase gene (*intI1*) of class 1 integrons of Gram negative bacteria (Alere Technologies GmbH, Jena, Germany) and the results interpreted by the ArrayMate, Alere.

## Results

### Isolation rates

The samples (n = 1984; dogs and cats) yielded a total 70 (3.53%, 95% CI: 2.72%–4.34%) KP and 23 (1.16%, 95% CI: 0.69%–1.63%) KO among the isolates, respectively. Of these, 15 (21.4%) KP and four (17.4%) KO revealed resistance to ESC and were investigated in this study.

### Genetic relatedness

**Figure 1:**
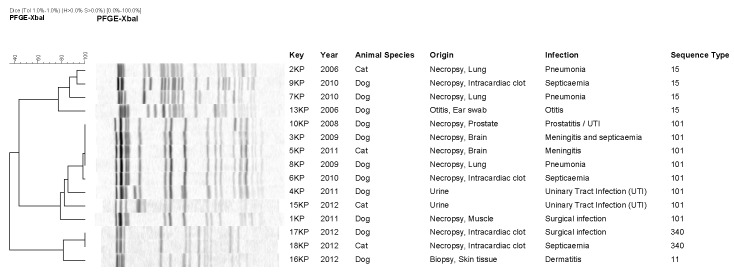
Dendrogram showing the genotypic relatedness of ESC-resistant *Klebsiella pneumoniae* (KP) isolates from dogs and cats based on *Xba*I-PFGE fingerprints, and comparison with Multilocus Sequence Typing classification.

**Figure 2:**
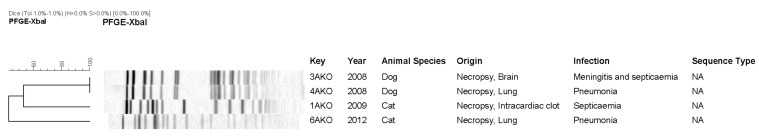
Dendrogram showing the genotypic relatedness of ESC-resistant *Klebsiella oxytoca* (KO) isolates from dogs and cats based on *Xba*I-PFGE fingerprints. Legend: NA =  Not Applicable.

The 15 KP isolates investigated by MLST were assigned to four different Sequence Types (ST): ST11 (n = 1), ST340 (n = 2), ST101 (n = 8), and ST15 (n = 4) (Figure 1). ST11 and its single-locus (*tonB*) variant (SLV) ST340 (3/15, 20%), both belonging to CC11, were detected in 2012. The separation of the isolates based on MLST corresponded well with PFGE results grouping the same isolates (Figure 1). A total of 11 different PFGE patterns were observed including two clusters of two and four indistinguishable isolates, respectively (Figure 1). The cluster of the two isolates both belonged to ST340 and was related (80% similarity) to a single isolate exhibiting a unique PFGE pattern and belonging to ST11. The other cluster of four indistinguishable isolates was highly related (from 99% to 80% similarity) to additional four isolates within the same PFGE group, all belonging to ST101 (Figure 1). No clustering was observed related to time, animal origin, nor infection, but some to the presence of resistance genes (Table 1). No MLST was assigned to the four KO isolates. However, the four isolates revealed three different PFGE patterns of which one was a cluster of two identical isolates (Figure 2). The three patterns seemed not to be related, indicating a similarity of 45% and 55% to the pattern of the two clustering isolates. Interestingly, the two isolates of the same PFGE pattern were both from dogs and isolated within the same year, but it could be the result of a random effect (Figure 2).

**Table 1:** Sequence Types, plasmid incompatibility groups, and antimicrobial resistance phenotypes and genotypes in ESC-resistant *Klebsiella pneumoniae* (KP) and *Klebsiella oxytoca* (KO).

Sequence Type	Key	Antimicrobial Resistance profile	Plasmid	ESBL and AmpC genes	PMQR genes	Other resistance and Integron genes
15	2KP	AMP,CTX,CFT,NAL,CIP,STR,KAN,CLO,SULFA,TRI,TET	IncI1; IncR	*bla* _CMY-2_, *bla*_SHV-28_		*aadA2; catA1; dfrA12; sul1; tet*(A)*; intI1*
15	9KP	AMP,CTX,CFT,NAL,CIP,STR,KAN,GEN,CLO,SULFA,TRI,TET	IncR; IncFIIk	*bla* _SHV-2a_, *bla*_SHV-28_, *bla*_TEM-1_		*strA/B; catA1; dfrA12; sulI; tet(A); intI1*
15	7KP	AMP,CTX,CFT,NAL,CIP,STR,KAN,SULFA,TRI	IncR	*bla* _CTX-M-15_, *bla*_SHV-28_, *bla*_TEM-1_	*aac(6′)-Ib-cr*	*strA/B; dfrA14; sulII; intI1*
15	13KP	AMP,CTX,CFT,NAL,CIP,STR,KAN,GEN,CLO,SULFA,TRI	IncN; IncFIA; IncFIB; IncR	*bla* _CTX-M-1_; *bla*_SHV-28_		*aadA2; strB; catA1; dfrA12; sulI; sulII*
101	10KP	AMP,CTX,CFT,NAL,CIP,KAN,GEN,SULFA,TRI,TET	IncR; IncFIIk; IncFII	*bla* _CTX-M-15_, *bla*_SHV-1_, *bla*_TEM-1_, *bla*_OXA-1_	*aac(6′)-Ib-cr*	*aac(6′)-Ib; dfrA17;*
101	3KP	AMP,CTX,CFT,NAL,CIP,KAN,GEN,TRI,TET	IncR; IncFIIk	*bla* _CTX-M-15_, *bla*_SHV-1_, *bla*_TEM-1_, *bla*_OXA-1_	*aac(6′)-Ib-cr*	*aac(6′)-Ib; aadA1; dfrA14; tet*(D)
101	5KP	AMP,CTX,CFT,NAL,CIP,KAN,GEN,TRI	IncR; IncFIIk	*bla* _CTX-M-15_, *bla*_SHV-1_, *bla*_TEM-1_, *bla*_OXA-1_	*aac(6′)-Ib-cr*	*aac(6′)-Ib; aadA1; dfrA14*
101	8KP	AMP,CTX,CFT,NAL,CIP,KAN,GEN,TRI,TET	IncR; IncFIIk	*bla* _CTX-M-15_, *bla*_SHV-1_, *bla*_TEM-1_, *bla*_OXA-1_	*aac(6′)-Ib-cr*	*aac(6′)-Ib; aadA1; dfrA14; tet*(D)
101	6KP	AMP,CTX,CFT,NAL,CIP,KAN,GEN,TRI,TET	IncHI2; IncR; IncFIIk	*bla* _CTX-M-15_, *bla*_SHV-1_, *bla*_TEM-1_, *bla*_OXA-1_	*aac(6′)-Ib-cr*	*aac(6′)-Ib; aadA1*
101	4KP	AMP,CTX,CFT,NAL,CIP,KAN,GEN,TRI,TET	IncR; IncFIIk	*bla* _CTX-M-15_, *bla*_SHV-1_, *bla*_TEM-1_, *bla*_OXA-1_	*aac(6′)-Ib-cr*	*aac(6′)-Ib; aadA1; dfrA14; tet*(D)
101	15KP	AMP,CTX,CFT,NAL,CIP,KAN,GEN,TRI,TET	IncR; IncFIIk	*bla* _CTX-M-15_, *bla*_SHV-1_, *bla*_TEM-1_, *bla*_OXA-1_	*aac(6′)-Ib-cr*	*aadA1; dfrA17; tet*(D)
101	1KP	AMP,CTX,CFT,NAL,CIP,STR,KAN,GEN,CLO,TRI,TET	IncHI2; IncR; IncFIIk	*bla* _CTX-M-15_, *bla*_SHV-1_, *bla*_TEM-1_, *bla*_OXA-1_	*aac(6′)-Ib-cr*	*aadA; strB; catA1; dfrA14*
340	17KP	AMP,CTX,CFT,NAL,CIP,KAN,GEN,SULFA,TRI,TET	IncHI2; IncR; IncFIIk, IncFII	*bla* _CTX-M-15_, *bla*_SHV-11_, *bla*_TEM-1_, *bla*_OXA-1,_ *bla*_DHA-1_	*aac(6′)-Ib-cr; qnrB; qnrS1*	*aadA2; dfrA21; dfrA17; sulI; tet*(A); *intI1*
340	18KP	AMP,CTX,CFT,NAL,CIP,KAN,GEN,SULFA,TRI,TET	IncR; IncFIIk	*bla* _CTX-M-15_, *bla*_SHV-11_, *bla*_TEM-1_, *bla*_OXA-1,_ *bla* _DHA-1_	*aac(6′)-Ib-cr; qnrB*	*aac(6′)-Ib; aadA4; dfrA17; dfrA19; sulI; tet*(A); *intI1*
11	16KP	AMP,CTX,CFT,NAL,CIP,SULFA,TRI,TET	IncN; IncFIIk	*bla* _CTX-M-1_, *bla*_SHV-11_, *bla*_TEM-1_	*qnrS1*	*aadA2; dfr12; dfrA1; sulI; tet*(A)
						
NA	3A KO	AMP,CTX,CFT,CIP,STR,KAN,GEN,SULFA,TRI,TET	IncHI2; IncL/M	*bla* _SHV-12_, *bla*_DHA-1_, *bla*_TEM-1_	*qnrB4*	*strA/B; dfrA19; sulI; tet*(D)*; intI1*
NA	4A KO	AMP,CTX,CFT,CIP,STR,KAN,GEN,SULFA,TRI,TET	IncHI2; IncL/M	*bla* _SHV-12_, *bla*_DHA-1_, *bla*_TEM-1_	*qnrB4*	*strA/B; dfrA19; sulI; tet*(D)*; intI1*
NA	1A KO	AMP,CTX,CIP,STR,SULFA,TRI,TET	IncHI2; IncP	*bla* _CTX-M-9_	*qnrA1*	*aadA2; aadB; sulI*
NA	6A KO	AMP,CTX,CIP,SULFA,TRI,TET	IncHI2	*bla* _CTX-M-9_	*qnrA1; aac(6′)-Ib-cr*	*aadA2; ant2la; sulI; intI1*

Legend:

NA: Not Applicable; AMP = Ampicillin; CFT = Ceftazidime; CIP = Ciprofloxacin; CLO = Chloramphenicol; CTX = Cefotaxime; GEN = Gentamicin; KAN = Kanamycin; NAL =  Nalidixic Acid; STR = Streptomycin; SULFA = Sulfamethoxazole; TET = Tetracycline; TRI = Trimethoprim.

Note: When underscored, plasmids and their content of beta-lactamase and PMQR genes where detected in transformant strains.

### Antimicrobial susceptibility testing

All isolates showed microbiological resistance to third-generation cephalosporins, and also clinical resistance, either when the MIC results were interpreted according to clinical breakpoints set by CLSI [16] or by EUCAST (e. g. MIC cefotaxime > = 4mg/L), except for 9KP (MIC 1 mg/L). The phenotype for PMQR was evident (ciprofloxacin MIC 0.25mg/L, nalidixic acid 8 mg/L) in KO isolates only, because in all KP isolates it was masked by concurrent genetic background conferring MICs of 8 and 128 mg/L, respectively.

Moreover, all isolates showed multidrug-resistance towards other classes of antimicrobials, such as aminoglycosides, sulfonamides, tetracyclines, dihydrofolate reductase inhibitors and amphenicols, mediated by *strA/B*, *aadA2*, *aadB*, *ant (2")-Ia*, *aac(6′)-Ib*, *sul*, *tet*, *dfr* and *cat* genes in various combinations, as reported in Table 1.

### Genes encoding ESBL-, AmpC-, and PMQR

All *Klebsiella* isolates investigated showed the presence of at least one ESBL or AmpC gene encoding ESC resistance. Additionally, 16 out of 19 isolates harbored a PMQR gene (*qnr* family or *aac(6′)-Ib-cr*, single or in combination). The most frequent ESC gene harbored by KP isolates was *bla*_CTX-M-15_ (n = 11, 58%), detected in all eight ST101, in one ST15 and in both two ST340 isolates, respectively. All four ST15 KP isolates carried the *bla*_SHV-28_ gene, single or in combination with the ESC resistance genes *bla*_CTX-M-15_, *bla*_CTX-M-1_, *bla*_CMY-2_, or with the *bla*_TEM-1_. All ST101 KP isolates harbored the variant of the *aac(6′)-Ib-cr* gene, encoding for PMQR. Similarly, the ST340 and ST11 KP isolates were the only ESC-resistant KP harboring genes within the *qnr* family.

The ST15, ST101 and ST340 KP isolates presented the *bla*_CTX-M-15_ gene, which was mostly associated with the *bla*_TEM-1_, *bla*_OXA-1_ and *aac(6′)-Ib-cr* genes, and to the *bla*_SHV-1_, or *bla*_SHV-11_ or *bla*_SHV-28_ (Table 1).

Of the four KO isolates, two were positive for *bla*_CTX-M-9_ gene, both associated with the PMQR gene *qnrA1*, with one the two also positive for the *aac(6′)-Ib-cr* gene. The last two KO isolates exhibited the *bla*_SHV-12_, *bla*_TEM-1_, *bla*_DHA-1_ and *qnrB4* genes. All isolates under study were negative for the presence of carbapenemase genes.

### Plasmid analysis

Plasmids detected from all the strains and classified by the PCR-based replicon typing method, are reported in Table 1.

In our study, the *bla*_CTX-M-15_ gene was successfully transferred by transformation from several MLST prototypic strains, demonstrating its plasmid localization. All but one transformant strains were positive for plasmids belonging to the IncR incompatibility group, and in the 18KP transconjugant the *bla*_CTX-M-15_ gene was co-transferred with the *bla*_DHA-1_ gene. Other ST15 KP strains also presented the IncR plasmid carrying different ESBL genes such as *bla*_SHV-2a_ and *bla*_CTX-M-1_. The 2KP ST15 strain harbored a IncI1 plasmid carrying the *bla*_CMY-2_ gene, while a IncN plasmid carrying both the *bla*_CTX-M-1_ and *qnrS1* gene was detected in the ST11 16KP strain.

The KO transformants carried the ESBL genes, *bla*_CTX-M-9_ and *bla*_SHV-12_ in two incompatibility groups, IncHI2 and IncL/M, respectively (Table 1).

## Discussion

The paper provides evidence of heterogeneity of determinants conferring ESC resistance in clinical *Klebsiella* isolates from dogs and cats in Italy. To the best of our knowledge, all the ESC resistant *Klebsiella* investigated were from sporadic clinical cases, although two clusters of KO (n = 2) and KP ST101 (n = 4) showed 100% similarity. Apparently, isolates were epidemiologically unrelated, with the exception of 6KP and 1KP (both ST101), with a DNA restriction profile showing 80% similarity, isolated from two cases of necropsies requested by the same veterinary practitioner in 2010 and 2011, respectively.

We also document the novel finding of the co-existence of the ESBL *bla*_SHV-28_ and the AmpC *bla*_CMY-2_ gene in one KP ST15 isolate from a cat and the first report of *qnrS*- and *qnrA*- and *aac*(*6*′)-*Ib-cr*- PMQR associated with *Klebsiella* infections in companion animals from Europe. *Klebsiella* with ESBL phenotype were described in dogs and cats from China [12], although it was mediated in those isolates by the presence of the CTX-M-9 and CTX-M-1 group beta-lactamases, while a CTX-M-15 positive ST15 KP clone was reported from hospital-acquired infections in pets from France [31]. In our study, the ESBL gene *bla*_CTX-M-15_ accounts for the majority of CTX-M genes detected in KP, but it was harbored mainly by the predominant KP ST101 lineage.

Interestingly, all KP ST15 isolates showed the association of the *bla*_CTX-M-15_, *bla*_CTX-M-1_, or *bla*_CMY-2_ genes with the *bla*_SHV-28_ gene, a *bla*_SHV-1_ mutant detected for the first time in China in 2002 (GenBank AF538324), and previously reported to encode for an ESBL phenotype [32]. Co-presence of *bla*_CTX-M-15_ and *bla*_SHV-28_ has been reported for the first time in the human KP ST15 epidemic clone by Nielsen et al., in 2011 [33]. Our transformation experiments did not succeed in transferring any of the *bla*_SHV28_ genes from positive KP isolates.

The multidrug-resistant CTX-M-15-producing KP isolates are an of important concern in the nosocomial infections and the IncFII-type plasmid is the main vehicle of *bla*_CTX-M-15_ transmission in human isolates [34]. However, in our study, this ESBL in KP from pets was mostly carried by IncR plasmids. The association of *bla*_CTX-M-15_ -IncR replicons in KP was documented for the first time by Coelho et al. in 2010 [35], in human clinical isolates, and also reported in the KP clone causing hospital-acquired infections in pets in France [31], and in Spain, associated to *qnrB4*, *bla*_DHA-1_ and *armA* genes. [36]

Interestingly, the 17KP transformant only presented the *bla*_CTX-M-15_ gene and the *qnrS1* gene both located in a IncFII plasmid. In our study, almost all the *bla*_CTX-M-15_ – positive isolates were also positive for IncFIIk replicons: although they have been specifically described in KP [30], these plasmids were never transferred in our experiments. Interestingly, in 2012 we have reported for the first time in clinical cases of pets from Italy the clone KP ST11 and its SLV ST340, harboring ESC and *qnrS1*-PMQR resistance. Among these CC11 isolates, the ST11 harbored IncN plasmid, which has been frequently involved in the transmission of the *bla*_CTX-M-1_ gene, a feature suggesting an animal reservoir for this ESBL, since this Inc plasmid types have been demonstrated to be highly prevalent in zoonotic enterobacterial pathogens [29]. The same animal origin reservoir is proposed for the IncI1 plasmids harboring the *bla*_CMY-2_ gene found in *E. coli* avian commensal strains [34].

It is noteworthy that ST11 and ST340 carried transferable ESBL resistance but not resistance to carbapenems. ST(CC)11 and ST15 and ST101 are among human epidemic clones, carrying both ESBLs and carbapenemases, which have been increasingly detected worldwide, in Europe and in Italy in the last years [36], [37], [38], [39], [40].

These infections are worrisome, since the antimicrobial treatment options for these multidrug-resistant strains are very limited. In Italy, during the last years the rapid emergence of the carbapenemase KPC-producing KP, belonging to the ST101, CC11, and predominantly to a single Sequence Type ST258, has become a serious problem in health-care settings [41], [42], [43].

As for CTX-M and SHV-12 ESBLs in Italy, a high occurrence in KP isolated from humans has been demonstrated, being the ST15, ST37, ST147 and ST273 the prevalent clones [44], [45], [46], [47].

In two KO isolates, the ESBL-encoding *bla*_SHV-12_ gene co-existed with the AmpC gene *bla*_DHA-1_ in accordance with phenotype of resistance to cefotaxime and cefoxitin observed in the ESBL phenotypic confirmatory test. These two isolates also carried the PMQR gene *qnrB4*. In these two KO transformants the IncL/M plasmid harbored both the *bla*_SHV-12_ and the *bla*_DHA-1_ genes but not the *qnrB4* gene. To our knowledge, this feature has never been described before.

The other KO presented the *bla*_CTX-M-9_ gene, located in a IncHI2 plasmid as described worldwide but associated to a *qnrA1* gene, a feature previously described in Spain in *E. coli* and KP of human origin [48], [49] and in KO in clinical specimens from Japan [50].

Similarly to what has been observed in other human and canine KP isolates [34], [12], the association of *bla*_CTX-M_ genes with the the *aac(6′)-Ib-cr* encoding an aminoglycoside acetyl transferase determining PMQR, was demonstrated in all ST101 and ST340 isolates, but only in one out of four ST15, but these PMQR genes were not located in the same plasmid in our strains. Conversely, PMQR encoded by different *qnr* genes of the *qnrA* or *qnrB* groups were observed in all the KO isolates studied (Table 1). In the case of the *qnrA1* gene, the two KO isolates also harbored the ESBL *bla*_CTX-M-9_ gene, a feature reported previously in association with *bla*_VIM-1_ and IncHI2 plasmids in KO of human origin [51].

Multidrug-resistance in the ESC resistant and PMQR isolates studied is of further concern from a therapeutic perspective, for a possible impact on clinical outcome of affected animals. In many isolates, the demonstration of the integrase *intI1*, accounts for the presence of resistance gene cassettes with *sul*, *aad*A, *cat*, *dfr* genes, associated with Class I integrons, similarly to what has been described in KP of human origin [52], [53]. As for streptomycin resistance, MIC>16 mg/L correlated in 100% isolates with the presence of *strA/B* genes.

Fortunately, the absence of carbapenemases offers so far a better scenario for antimicrobial therapy in companion animals, although a possible circulation, within a short time, of these carbapenemase-producing epidemic strains, is of concern also in veterinary medicine.

In conclusion, monitoring and characterization of multidrug-resistant *Klebsiella* in companion animals by means of phenotypic and molecular methods proved to be useful for providing a picture of mechanisms of resistance that may further spread clonally or by horizontal gene transfer, at regional or even at international level. Sharing this kind of information appears essential for building awareness in companion animal therapy, also in view of preventing and controlling the spread of multidrug-resistant strains in veterinary hospital settings. Indeed, the bi-directional exchange between owners and pets of *Klebsiella* carrying resistance to critically important antimicrobials for human health, raise some concerns also for the possibility of a spill back to humans, especially at household level.

The emergence of the PMQR and, above all, the emergence of concurrent transferable cephamycin, oxymino-cephalosporin, and beta-lactamase inhibitor resistance in multidrug-resistant *Klebsiella* isolates in pets, a recent issue even in human therapy [54], may pose in the next future further and serious therapeutic challenges also in bacterial infections of companion animals.
